# Cumulative violence exposures among men who have sex with men living with HIV in India: Psychosocial correlates of HIV care continuum outcomes

**DOI:** 10.1371/journal.pone.0295225

**Published:** 2023-12-01

**Authors:** Bushra Sabri, Chakra Budhathoki, Allison M. McFall, Shruti H. Mehta, David D. Celentano, Sunil S. Solomon, Aylur K. Srikrishnan, Santhanam Anand, Canjeevaram K. Vasudevan, Gregory M. Lucas

**Affiliations:** 1 School of Nursing, Johns Hopkins University, Baltimore, MD, United States of America; 2 Bloomberg School of Public Health, Johns Hopkins University, Baltimore, MD, United States of America; 3 Division of Infectious Diseases, School of Medicine, Johns Hopkins University, Baltimore, MD, United States of America; 4 YR Gaitonde Centre for AIDS Research and Education (YRGCARE), Chennai, India; University of Toronto, CANADA

## Abstract

Lifetime exposures to violence among men who have sex with men (MSM) are associated with multiple psychosocial health risks and can affect engagement and outcomes of HIV treatment. This study a) explored relationships between levels of exposures to violence and HIV care continuum outcomes among MSM living with HIV in India, and b) identified psychosocial correlates of HIV care continuum outcomes among MSM living with HIV and those with lifetime cumulative exposures to violence (CVE). CVE referred to exposures to violence in both childhood and adulthood. This cross-sectional analysis used survey data collected between August 2016 and May 2017 from 1763 men who have sex with men living with HIV across 10 cities in India, using respondent-driven sampling. We found that higher levels of violence exposure were significantly associated with lower awareness of HIV positive status, and lower likelihood of initiating antiretroviral therapy. Compared with MSM living with HIV that had no CVE, those with CVE were more likely to report perpetration of interpersonal violence, alcohol misuse, depressive symptoms, and HIV transmission risk behaviors and to have two to four co-occurring psychosocial problems. In multivariable analysis with the subset of MSM with CVE, psychosocial correlates significantly associated with at least one HIV care continuum outcome were HIV transmission risk behaviors, perpetration of interpersonal violence, depression, and alcohol misuse. The findings highlight the need for integrating care for lifetime violence exposures and associated behavioral problems in HIV care settings for men who have sex with men living with HIV in India.

## Introduction

Violence against men who have sex with men (MSM) is a significant public health and human rights concern. Research globally demonstrates that sexual minorities, including MSM, are more likely to be survivors of interpersonal violence than the general population [1–5]. Interpersonal violence experiences among MSM can include experiences of physical, sexual and/or psychological abuse in childhood, adolescence and/or adulthood by any perpetrator [1, 3, 6]. Violence victimization is common among MSMs in India [6, 7]. In our prior study of 11,788 MSM in 12 cities in India, 22.4% of MSM reported child sexual abuse [8]. Further, those who are living with HIV are likely to have disproportionately high levels of violence exposures in childhood and adulthood [9].

MSM face violence from family and community, often due to their sexual identity and barriers to socio-cultural acceptance of sexual minority individuals. Violence, however, is likely to be under-reported due to the marginalization of MSM in India and sanctions against same-sex relationships [10]. Lifetime violence is a key factor contributing to HIV-related disparities among MSM [11]. In India, MSM are disproportionately affected by the HIV epidemic [12], with both the prevalence and incidence of HIV being disproportionately higher among MSM than the general population [13]. This may be due to additive effects of syndemic psychosocial conditions (e.g., depression, drug use, HIV risk behaviors) [11, 14]. According to the syndemic theory [15, 16], multiple co-occurring conditions (e.g., mental health (MH), substance use, violence, HIV risk behaviors) can worsen the disease burden on a population and amplify the negative consequences of the individual conditions. Therefore, there is need to examine the effects of syndemic conditions on HIV care continuum outcomes among (MSM living with HIV) MSMLWH with violence exposures in India.

Violence is significantly associated with poor MH outcomes among MSM. In our prior study, violence victimization (i.e., forced sex), MSM-related stigma, and, among those who were HIV positive, awareness and disclosure of HIV status to another were associated with higher odds of depression [17]. Further, rates of MH problems (e.g., depression) are higher among both people vulnerable to acquiring HIV and people living with HIV. Poor MH has been linked to poorer adherence to HIV treatment, increased viral load and decreased CD4 counts among HIV positive individuals [9, 18]. Fear of violence, can be a barrier to disclosure of HIV status, accessing HIV care, and adherence to medication [9, 17]. Exposures to violence may also negatively affect immune function, leading to faster progression of HIV disease [9, 19]. Health impacts of violence may be incrementally worse for HIV positive MSM with cumulative violence exposures (CVE).

CVE are multiple types of interpersonal violence exposures (e.g., physical, sexual) from multiple perpetrators across the lifespan. CVEs have been associated with poor mental (e.g., depression) [20], physical (e.g., HIV) and behavioral health outcomes [21], including for people living with HIV. Among MSMLWH, violence, and its MH effects (e.g., psychological distress, internalized stigma) may compound the stress of managing HIV, and interfere with engagement in HIV care, leading to poor outcomes such as lower ART use, poorer adherence to HIV care, and non-suppressed viral load [21]. Mental health and substance abuse issues have been identified as major barriers to viral suppression among MSMLWH in India [22]. Thus, MSMLWH with CVE may be less likely to be engaged in care, access to or adhere to HIV treatment, or be virally suppressed than those with fewer or no violence exposures. However, there is a gap in research on psychosocial correlates of HIV care continuum outcomes among MSMLWH with CVE in India and other low-and middle-income countries. Therefore, drawing from the syndemic theory [17, 18], this study examined psychosocial correlates of HIV care continuum outcomes among MSMLWH with CVE in India.

## Materials and methods

### Participants and procedures

We analyzed cross-sectional survey and laboratory data from 1763 MSMLWH in India recruited using respondent-driven sampling (RDS) from 10 cities between August 2016 and May 2017. The sample for analysis excluded RDS seeds. The RDS survey was conducted as the evaluation phase of a cluster-randomized trial that evaluated the effect of integrated care centers on HIV testing frequency among MSM [23]. RDS is a sampling method that is widely used to characterize the epidemiology of “hidden” populations. Application of RDS sampling weights produces unbiased estimates of characteristics of interest in the target population [24]. Eligibility criteria included: (1) age ≥18 years, (2) cisgender men (transgender women/hijras were excluded), (3) oral/anal sex with a man in the prior 12 months, and (4) provided oral consent. Following oral consent, all participants completed a 45- to 60-minute interviewer-administered survey that covered demographics, sexual identity, risk behaviors, drug and alcohol use, stigma, violence exposures, depression, and access to HIV-related and general health services. All participants were tested for HIV antibodies using three rapid tests using the following kits: Alere^TM^ Determine^TM^ HIV-1/2 (Alere Medical Co., Ltd., Chiba, Japan); First response HIV card test 1–2.0 (PMC Medical India Pvt Ltd, Daman, India); Signal Flow Through HIV 1+2 Spot/Immunodot Test kit, (Span Diagnostics Ltd, Surat, India). All HIV antibody positive samples were subsequently tested for HIV RNA. HIV testing was accompanied by pre- and post-test counseling; for those that tested positive, CD4 cell counts were measured by flow cytometry and HIV RNA was measured by RealTime HIV-1 (Abbott Laboratories, Abbott Park, IL, USA). The study was approved by the institutional review boards of YRGCARE in Chennai, India, Johns Hopkins Medicine and Johns Hopkins Bloomberg School of Public Health in Baltimore, Maryland, USA.

### Measures

#### Demographic variables

***Age*** was measured as a continuous variable. ***Education*** was categorized into primary school or less, secondary school and high school or beyond. ***Marital status*** categories included never married, currently married/living with partner and widowed, divorced or separated. ***Employment*** categories included monthly wages, weekly/daily wages, unemployment and other. For ***financial stress***, participants reported how often in the last 12 months they ran out of money for necessities categorized as at least monthly and never or less than monthly.

#### Other relevant variables

***Social support*** was measured using five items from the Social Support Scale [25–27] that assessed how often participants had different kinds of support available to them during the past 4 weeks. The items were rated using a 4-point scale ranging from 0 (none of the time) to 4 (all of the time), with Cronbach’s alpha of 0.892. Categories include low (<10), medium (11–19) and high (20–25). For ***stigma*,** a composite stigma score was created by averaging stigma scores from four scales [28] adapted for our needs (i.e., stigma related to being an MSM): vicarious stigma, enacted stigma, felt normative stigma and internalized stigma. The response options for items ranged from 0 = not at all to 3 = a great deal. The composite scores range from 0 to 20 with higher scores on the measure indicating more stigma [19], with overall Cronbach’s alpha of 0.957.

#### Psychosocial factors

These included: ***Depressive symptoms*:** The Patient Health Questionnaire (PHQ-9; 9 items) was used to assess for depression symptoms within the past two weeks. The response options ranged from 0 (not at all) to 3 (nearly every day) with total scores ranging from 0 to 27. Higher scores on the PHQ-9 indicate higher levels of depressive symptoms. The presence of depression was measured using a cut-off score of 10 or higher [29]. ***Sexual HIV transmission risk behaviors*** were measured using a) ***Unprotected sex***, assessed based on participant engaging in any unprotected sex with any sex/gender in prior 6 months. b) ***Exchange sex***, assessed based on participants ever engaging in sex to receive money, alcohol, or other things within the past six months. c) ***Multiple male and transgender sex partners***, assessed based on how many different men or hijra have participants had anal sex with in the past 6 months.

***Alcohol use problem*** was measured using the Alcohol Use Disorders Identification Test, a measure for hazardous alcohol use. The score of 15 or higher on the measure indicate alcohol dependence [30, 31]. ***Substance use*:** For drug use problems within the past six months, participants were asked if they injected drugs for non-medicinal purposes or if they used any drug for non-medicinal purposes by a non-injection route [32].

***Perpetration of physical or sexual violence*:** For perpetration of physical or sexual violence, participants were asked if they ever tried to make someone have sex when they did not want or if they ever hit, slapped, kicked, pushed, shoved, or otherwise physically hurt a sexual partner.

#### Violence victimization experiences

For interpersonal violence exposures, participants were asked about their experiences of physical and sexual violence in childhood and/or adulthood. Example item included- “when you were growing up (before 16 years old), did you experience any serious physical violence? Level of violence exposures included three categories: 0 = no violence, 1 = violence during one stage of life only, either childhood or adulthood, and 2 = cumulative exposures to violence. Cumulative violence exposure was measured by summing the affirmative responses to the exposures to violence in both childhood and adulthood.

#### HIV care continuum outcomes

HIV care continuum outcomes included awareness of HIV positive status, linkage to care, initiation of ART and viral suppression. All outcomes were self-reported except for viral suppression. Awareness was defined as a prior HIV diagnosis or being told previously they had HIV with HIV status determined based on HIV testing at the study visit (described above). Linkage to care was defined as ever visiting a care provider for management of HIV after diagnosis. ART initiation was measured based on self-reported initiation of ART and viral suppression was measured based on plasma HIV RNA <150 copies/ mL.

### Data analysis

We analyzed data from a sub-sample of 1763 MSMLWH, using bivariate and multivariable analysis procedures. Categorical variables were summarized using frequency and percentages. Continuous variables were summarized using median and inter-quartile range or mean and standard deviation as appropriate. Four HIV care continuum outcome variables, all dichotomous, were analyzed: awareness of HIV positive status, linkage to care, initiation of ART, and viral load suppression. For analysis of associations with HIV outcomes, participants who did not complete the previous step were excluded. For example, the analysis of ART initiation only included those who were linked to care. To compare sample characteristics across the three violence categories, chi-square test, and Kruskal-Wallis test were run respectively for categorical variables and continuous variables. Binary logistic regression models were used to evaluate the association between the HIV care continuum outcome variables and independent variables adjusting for potential covariates. First, bivariate models were used to screen candidate predictor variables for further multivariable analyses. Then the predictors that had a *p*≤0.15 were included in multivariable analyses using a backward selection method. One final model for each HIV care continuum outcome variable was selected to identify predictors of the outcome. To study characteristics of participants who experienced CVE, the sub-sample of participants who experienced both types of violence (childhood and adulthood, *n* = 323) were also analyzed using the similar bivariate and multivariate methods. We performed both unweighted and weighted analyses. The weighted analyses used network size as weights, using the RDS-II estimator (Volz-Heckathorn estimator) [24]. All the data management and analyses were performed using Stata and SAS.

## Results and discussion

Participants included in the analysis were 1763 MSMLWH, with a median age of 36 years (IQR = 29, 41). Most MSM identified as *kothi* (receptive or who were penetrated in the sexual encounter)) (43.6%, *n* = 767), followed by double decker (receiver as well as penetrator) (27.4%, *n* = 482), *panthi* (i.e., penetrator) (22.4%, *n* = 395) and gay or bisexual (6.6%, *n* = 117). Most of the MSMLWH were married or living with a partner (68.8%, *n* = 1212), with only 54.4% (*n* = 741) reporting disclosure of HIV positive status to a spouse or primary partner. Forty-two percent (*n* = 736) of them had completed secondary school and 29.4% (*n* = 519) graduated from high school or completed higher education. More than half of them were employed, engaged in monthly (45.8%, *n* = 807) or weekly/daily wage (44.0%, *n* = 776) work. Forty-three percent (*n* = 763) reported financial stress. More detailed summaries of the sample characteristics are presented in Table 1. The sections below present weighted findings.

**Table 1:** Characteristics of MSM living with HIV by level of violence exposures (weighted).

% or median (25^th^-75^th^ percentiles)	No history of violence *n* = 1060	Violence in childhood/ adulthood only *n* = 380	Both childhood and adulthood violence *n* = 323	*p-*value	Total *n* = 1763
*Age*	37 (29, 41)	35 (27, 41)	36 (28, 42)	0.003	36 (29, 41)
*Sexual identity*				<0.001	
** Panthi**	31.6%	17.3%	11.0%		26.0%
** Kothi**	27.2%	35.1%	45.2%		31.9%
** Double Decker**	30.5%	35.0%	27.0%		31.0%
** Gay / Bisexual / MSM**	10.6%	12.5%	16.9%		11.8%
*Marital Status*				<0.001	
** Never married**	22.9%	42.3%	38.6%		29.1%
** Currently married/living with partner/long-term partner**	73.0%		55.4%		66.3%
** Widowed, divorced/ separated**	4.1%	52.6%	6.0%		4.6%
*Employmen*t				0.397	
** Monthly wages**	56.1%	53.9%	48.5%		54.7%
** Weekly/daily wages**	33.7%	33.2%	39.4%		34.1%
** Unemployed or laid off**	3.9%	4.8%	3.9%		4.0%
** Other**	6.3%	8.1%	8.2%		6.6%
***Education***				0.144	
** Primary school or less**	23.1%	21.6%	22.6%		22.7%
** Secondary school**	41.8%	37.4%	35.8%		40.1%
** High school and above**	35.1%	41.0%	41.7%		37.2%
*Financial Stress*	32.7%	52.3%	42.4%	< .001	38.3%
*Psychosocial Conditions*					
**Alcohol Problems**	35.6%	38.2%	53.9%	< .001	38.4%
**Ever Injected Drugs**	2.2%	3.1%	4.0%	0.261	2.6%
**Any drug use in prior 6 months (Injection or non-injection)**	2.9%	4.5%	4.9%	0.157	3.5%
**Number of male sexual partners (Prior 6 months)**	1 (0, 2)	1 (1, 4)	3 (1, 6)	< .001	1 (0, 3)
**Unprotected Sex (Prior 6 months)**	18.1%	14.4%	25.3%	0.004	18.1%
**Exchange Sex (Prior 6 months)**	11.1%	30.6%	36.9%	< .001	18.6%
**Perpetration of violence**	3.4%	30.1%	55.0%	< .001	15.6%
**Depression, median (Q1, Q3)**	3 (0, 7)	4 (1, 8)	5 (2, 9)	< .001	4 (0, 7)
**Depression, n (%)**	11.4%	17.9%	22.6%	< .001	14.2%
**Stigma median (Q1, Q3)**	4.3 (1.3, 7.3)	6.0 (3.6, 8.4)	5.7 (4.2, 8.5)	< .001	4.8 (2, 7.6)
^*1*^*Number of co-occurring psychosocial problems* (e.g., depression, alcohol misuse etc.)					
**Median (Q1, Q3)**	1 (0, 1)	1 (0, 2)	2 (1, 2)	< .001	1 (0, 1)
**No psychosocial problems**	45.9%	33.2%	13.5%		39.2%
**One psychosocial problem**	39.8%	38.5%	36.5%		39.1%
**Two co-occurring problems**	12.0%	20.7%	29.6%		16.1%
**Three co-occurring problems**	2.1%	5.4%	15.7%		4.5%
**Four co-occurring problems**	0.2%	2.2%	4.6%		1.2%
**Five co-occurring problems**	0.1%	0.1%	0.0%		0.1%
*HIV Care Continuum*					
**Aware of HIV-positive Status**	78.8%	74.7%	70.6%	0.017	76.9%
**Linked to Care**	93.0%	87.0%	91.7%	0.005	91.6%
**Initiated ART**	91.4%	86.8%	84.9%	0.014	89.7%
**Virally Suppressed**	78.0%	76.7%	73.6%	0.553	77.3%

^1^ n = 1361

^1^Co-occurring conditions refer to co-occurrence of the following psychosocial condition: Depression, alcohol problem (i.e., hazardous alcohol dependence), any drug use problem (prior 6 month), violence perpetration (physical or sexual), HIV risk behavior (i.e., unprotected sex with me or women in prior 6 months).

### Univariate and bivariate analysis (*n* = 1763)

#### Psychosocial factors among MSMLWH with different levels of violence exposures

Table 1 presents characteristics of MSMLWH by level of violence exposure. Twenty-two percent (*n* = 380) of MSMLWH reported exposure to violence in either childhood or adulthood, and 18.3% (*n* = 323) had CVE (i.e., violence in both childhood and adulthood). A significantly greater percentage of MSMLWH with CVE (55%) reported perpetration of physical or sexual violence than the MSMLWH with no histories (3.4%) or one type of violence exposure (30.1%, *p* < .001 for overall comparison). Almost half of the MSMLWHI with CVE identified as kothi (45.2%) and were currently married or living with a long-term partner (55.4%).

MSMLWH with CVE had the highest percentage of hazardous alcohol use or dependence (54%), when compared to those with no history of violence (36%) or violence in only childhood or adulthood (38.2%) only *(p* < .001). Compared to the other groups, unprotected sex *(p* < .01) and exchange sex *(p* < .001) in the prior 6 months was also more prevalent among the MSMLWH with CVEs than among those in the one type or no violence categories. Further, there were significant differences in scores on depression symptoms across the violence groups (*p* < .001). MSMLWH with CVE had higher median scores on depression (*Median* = 5) than those with exposure to violence in either childhood or adulthood (*Median* = 4) and significantly higher than those with no histories of violence (*Median* = 3) (*p* < .001) Co-occurring psychosocial factors (i.e., MH, alcohol/drug use, sexual risk behaviors and violence) varied among MSM LWH with various levels of exposures to violence. Increasing exposure to violence was associated with larger numbers of psychosocial problems. The most common co-occurring problems among MSM LWH with CVEs were depression, alcohol problem, perpetration of violence and HIV transmission risk of unprotected sex.

#### Relationship between violence exposures and HIV care continuum outcomes

In bivariate analyses, violence exposure had significant associations with three of the four HIV care continuum outcomes (Table 1). Among the analytic cohort of MSM LWH, 78.8%, 74.7%, and 70.6% were aware of their HIV-positive status among those with no violence history, violence exposure in one stage of life, and CVE, respectively (*p* = 0.017 for overall comparison). Among the subset who were aware of their HIV-positive status, linkage to care (*p* = 0.005) and ART initiation (*p* = 0.014) remained significantly associated with violence exposure with MSMLWH with CVE less likely to be linked to care than those with no histories of violence. MSMLWH with CVE were also less likely to initiate ART than those with no histories of violence and those with violence in one stage of life (Table 1).

#### Multivariable analysis (n = 1763)

Using potential predictors of HIV care outcomes based on significance of bivariate analysis results, we ran multivariable analysis for each HIV care outcome. We found that exposure to any or both types of violence were not significant predictors of awareness of HIV positive status and linkage to care after controlling for covariates. However, exposure to any type of violence was a significant predictor of ART initiation after controlling for age, financial stress, unprotected sex, perpetration of physical or sexual violence, alcohol use, depression, and social support. The odds of ART initiation were 50% less (*AOR* = 0.50, 95% *CI*: 0.30, 0.85) for those who experienced violence in either childhood or adulthood compared to those who did not experience any violence. The comparison of both types of violence vs. no violence was not significant for ART initiation.

#### Results of multivariable analysis of psychosocial correlates of HIV care continuum outcomes among MSMLWH with CVE (n = 323)

We then performed bivariate and multivariable analysis of the HIV care continuum outcomes and the characteristics that were likely to be associated in the sub-sample of MSMLWH with CVE that we would expect to have poor HIV care outcomes. Similar to previous sample of n = 1763, we identified potential covariates using bivariate analyses in this sub-sample of *n* = 323, and then selected a multivariable model for each HIV care continuum outcome. Among MSMLWH with CVE, HIV transmission risk behaviors and perpetration of violence had significant associations with HIV care outcomes. Awareness of HIV status was significantly lower among those who engaged in sex work (*AOR =* 0.43, *CI =* 0.22–0.82) and reported unprotected sex (*AOR =* 0.08, *CI =* 0.04–0.18), but was higher among those with depressive symptoms (*AOR* = 4.10; *CI* = 1.57–10.68). Among those aware of their status, linkage to care was significantly lower among those with a history of perpetration of physical or sexual violence (*AOR =* 0.22, *CI =* 0.06–0.88), alcohol problems (*AOR =* 0.08, *CI =* 0.02–0.38) and experiences of stigma (*AOR =* 0.79, *CI =* 0.69–0.73–0.87–0.92). Further, viral suppression was significantly lower among MSMLWH with CVE and comorbid alcohol misuse problems (*AOR* = 0.45, *CI* = 0.22–0.90) (Table 2).

**Table 2:** Correlates of HIV care continuum outcomes among MSM with cumulative exposures to violence and living with HIV (*n* = 323) (weighted).

	Awareness of HIV positive status	Linked to Care	Initiated ART	Suppressed Viral Load
	*OR* (95% *CI*)	*OR* (95% *CI*)	*OR* (95% *CI*)	*OR* (95% *CI*)
	(*n* = 323)	(*n* = 235)	(*n* = 212)	(*n* = 175)
***Demographic Variables***				
***Age***	**1.09 (1.04, 1.13)**	-	1.07 (1.00, 1.14)	**1.08 (1.03, 1.13)**
***Education***				
** Primary school/less**	**-**	-	Ref	-
**Secondary school**			0.25 (0.06, 1.13)	
** High school & above**			0.59 (0.12, 2.89)	
*Employment*				
** Monthly wages**	0.43 (0.07, 2.67)	**-**	-	-
** Weekly/daily wages**	**0.13 (0.02, 0.78)**			
** Unemployed/laid off**	Ref			
** Other**	**0.08 (0.01, 0.63)**			
**Ran out of money for basic necessities**	-	-	-	**2.67 (1.16, 6.14**)
*Psychosocial Conditions*				
***Number of Male Sexual Partners***	-	-	-	0.99 (0.97, 1.00)
***Unprotected sex with any sex/gender in prior 6 months***				
**Always protected sex**	Ref	-	Ref	-
**Unprotected sex**	**0.08 (0.04, 0.18)**		**0.18 (0.04, 0.77)**	
**No sexual partners**	3.68 (0.84, 16.11)		0.43 (0.09, 1.98)	
***Engagement in sex work-prior 6 months***	**0.43 (0.22, 0.82)**	-	-	-
***Perpetration of violence***	-	**0.22 (0.06, 0.88)**	-	1.85 (0.95, 3.61)
***Any drug use***		**-**	**-**	**-**
***Alcohol Use***	-	**0.08 (0.02, 0.38)**	**-**	**0.45 (0.22, 0.90)**
***Stigma***	-	**0.79 (0.69, 0.92)**	-	1.10 (0.99, 1.22)
***Depression***	**4.10 (1.57, 10.68)**	-	0.34 (0.12, 1.01)	-

Note: Odds ratios that are significant at 5% are in bold in multivariable model.

### Discussion

Among a multi-city sample of MSMLWH in India, we found that violence exposure was associated with worse performance on 3 of 4 HIV care continuum steps in unadjusted analyses, with violence remaining associated with ART initiation in the adjusted analysis among all MSMLWH. Our findings showed that compared to those with no history of violence or violence victimization in one stage of life only, a lower percentage of MSMLWH with CVE were aware of HIV positive status or initiated ART. Further, a greater proportion among MSMLWH with CVE reported psychosocial problems and two to four co-occurring psychosocial problems. MSMLWH with CVE also had higher scores on depression than those with other levels of violence exposures. In prior research, greater exposures to violence have been associated with multiple co-occurring psychosocial issues such as depression, substance use, and HIV risk [21]. Co-occurrence of these issues also increases the likelihood of disparities in HIV prevention and care, thereby increasing disease burden for key populations [21] such as MSM. The effects of these co-occurring psychosocial problems on HIV care outcomes increase with each additional condition (e.g., depression), such that every increase in condition is associated with higher viral load [21, 33]. Thus, MSMLWH with CVE are a high-risk group with more challenges associated with engagement in HIV care and achieving viral suppression. Therefore, focusing on MSMLWH with CVE, this study identified psychosocial factors (e.g., alcohol/drug use, perpetration of violence) associated with HIV care outcomes among MSMLWH with CVE.

We found that MSMLWH with CVE who were engaged in HIV transmission risk behaviors were significantly less likely to be aware of their HIV positive status. This shows that MSMLWH with CVE need more attention in terms of testing for HIV, making them aware of their HIV status and preventing continued transmission of HIV to their sexual partners. Risky sexual behaviors among MSMLWH pose a risk to spouses as well as other sexual partners. In a prior study, the strongest predictor of HIV prevalence among wives of MSM was husband’s positive status [34]. Traumatic life experiences and co-occurring issues (e.g., stigma) heighten the risk for risky sexual behaviors [35] and the risk for alcohol/drug misuse to cope [36]. Alcohol/drug misuse and associated loss of inhibition and impaired judgement can further increase the likelihood of engaging in risky sexual behavior among MSMLWH [37, 38]. These factors also reduce the likelihood of accessing HIV care services. Thus, interventions are needed to increase regular and frequent HIV testing among MSM with CVE, specifically targeting those engaged in exchange sex and with multiple non-regular partners. Interventions are also needed to promote condom use among MSM, especially those with histories of violence to prevent infection and transmission of HIV.

In our study, alcohol use among MSMLWH with CVE was found to be related to significantly lower likelihood of linkage to care and suppressed viral load. In prior studies with other populations [39, 40], greater alcohol use was found to be associated with poor HIV care continuum outcomes among those who were HIV positive [40]. Alcohol use may interfere with active engagement in HIV treatment which in turn may influence viral suppression. Although additional research is needed to explore mechanisms underlying associations between alcohol use and HIV care outcomes, it is possible that associations are influenced by complex factors at multiple levels (e.g., community and individual) [40]. For example, impaired decision making after alcohol use and forgetfulness may impact adherence to regular HIV treatment [39]. These findings call for integrated violence and addiction treatment interventions in HIV care settings to improve HIV care outcomes for MSMLWH with CVE. Integrated interventions in HIV care settings can increase the proportion of HIV positive individuals achieving viral suppression, achieve treatment as prevention mandates and reduce risk for HIV transmission [39].

Exposure to violence is a risk factor for perpetration of violence [41], which can be a barrier to care engagement. In our study, perpetration of physical or sexual violence among MSMLWH with CVE was associated with lower odds of linkage to care. One explanation could be co-occurrence of violent behaviors with MH and substance abuse issues (e.g., alcohol misuse) [42]. Other explanations could be stress because of living with HIV, poor social relationships, discrimination from the stigma and being banned from social settings due to violent behaviors, which could all act as barriers to linkage to care. We also found stigma as a significant factor in linkage to care, with an increase in stigma associated with lower likelihood of linkage to care among MSMLWH with CVE. This is supported by studies in other countries where stigma remains a barrier to HIV treatment [43]. Particularly, internalized stigma and associated depressive symptoms are related to lower levels of care engagement among MSMLWH [21, 43]. Thus, interventions that address emotional issues related to victimization and perpetration and stigma associated with sexual minority status and being HIV-infected might enhance care engagement among MSMLWH in India.

MSMLWH with CVE had significantly higher scores on depression than the other MSMLWH with no histories of violence or those with one type of violence exposure. Studies have shown a strong association between lifetime exposures to violence and depression [20, 44]. Among HIV care continuum outcomes, depression was only significantly associated with awareness of HIV positive status. Although depression was not found to be significantly associated with other HIV care outcomes after adjusting for covariates in our study, it has been found to be a significant barrier to initiating ART, discontinuing ART or HIV treatment non-adherence in other studies [45–47]. Thus, there is need to address violence as well as depression issues within HIV care settings. Integration of violence and MH screening and care in HIV care settings would strengthen HIV care outcomes [47] for MSMLWH with CVE. Specifically, interventions to reduce depression symptoms may support HIV care continuum outcomes for MSM LWH with traumatic life experiences.

The present study has some limitations. First data was based on self-report, except for viral suppression. Second, the cross-sectional design makes it difficult to establish causal relationships between the studied variables. Despite the limitations, the strength of the study is a large community-based sample of MSM across multiple sites in India.

## Conclusion

Violence victimizations of MSMLWH are associated with multiple psychosocial health risks and can impact engagement and outcomes of HIV care. There is need for trauma-informed multicomponent interventions addressing trauma related to exposures to violence and associated health issues among MSMLWH in HIV care settings in India. Violence screening and support must be integrated in routine care for MSMLWH whose co-occurring issues may be interfering with HIV care engagement. Moreover, community-level efforts are needed to prevent violence, and reduce stigmatization and marginalization of MSM populations in India.
